# Cartography: Innateness or Convergent Cultural Evolution?

**DOI:** 10.3389/fpsyg.2022.887670

**Published:** 2022-04-25

**Authors:** Deniz Satık

**Affiliations:** Department of Linguistics, Harvard University, Cambridge, MA, United States

**Keywords:** cartography, left periphery, convergent cultural evolution, universal grammar, syntax, language faculty

## Abstract

Haspelmath argues that linguists who conduct comparative research and try to explain patterns that are general across languages can only consider two sources of these patterns: convergent cultural evolution of languages, which provides functional explanations of these phenomena, or innate building blocks for syntactic structure, specified in the human cognitive system. This paper claims that convergent cultural evolution and functional-adaptive explanations are not sufficient to explain the existence of certain crosslinguistic phenomena. The argument is based on comparative evidence of generalizations based on Rizzi and Cinque's theories of cartographic syntax, which imply the existence of finely ordered and complex innate categories. I argue that these patterns cannot be explained in functional-adaptive terms alone.

## 1. Introduction

One of the most controversial topics in the scientific study of language is whether there is language-specific innate cognitive machinery. While no one doubts that humans have a unique capacity for language compared to other animals, scientists disagree on whether an innate capacity for language—which Chomskyan linguists call “universal grammar” or the “language faculty”—exists. Haspelmath (2020) believes that it was premature for generative linguists to conclude that there is an innate capacity for language. He suggests that there can only be two sources of patterns across languages which are not due to historical accidents:

(i) convergent cultural evolution of languages to the same needs of speakers,
(ii) constraints on biologically possible language systems: innate building blocks (natural kinds) that provide a rigid blueprint for languages.

The key claim is that, if one allows for cultural or functional explanations for patterns seen from language to language, much, if not all, of the observation for the appeal to innate linguistic capacity would disappear. Haspelmath claims that the evidence from comparative linguistics, at this point in time, does not provide evidence in favor of an innate blueprint.

The goal of this article is to meet Haspelmath's challenge. I do not challenge Haspelmath's reasoning that linguists must take one of the two paths he describes above, which for the purposes of this article I concur with. I provide a survey of crosslinguistic evidence that has been provided in the generative grammar framework which might indicate the presence of innate machinery for syntactic structure. Although it is less challenging from an evolutionary standpoint for languages to have evolved culturally rather than biologically, I will discuss recent research which could indicate the presence of innately ordered syntactic categories, concluding that there cannot be a functional explanation for them.

## 2. Crosslinguistic Convergence Due to Convergent Cultural Evolution?

In this section, I introduce the reader to Haspelmath (2020), to lay the foundation for the view that I will argue against in this article. I will provide the reader to the basic concepts behind the debate at hand before moving on to discussing potential evidence in favor of there being innate building blocks for natural language.

There is no doubt regarding the unique and biological human capacity for language, which all scientists agree on. But what is controversial is how to *account* for this capacity, for which cognitive scientists split into two camps. Generative linguists belong to the first camp: to put it as broadly as possible, generative linguists believe that there is a there is an innate system of mechanisms and principles that are unique to humans which is used for language acquisition. Chomsky (2000) calls this innate system a “language organ,” and for generative linguists, he suggests that it is an object of study in the same way that biologists study literal organs like the heart or the kidneys^1^.

There is, however, little agreement between generative linguists regarding the nature of this innate faculty. Barsky (2016) takes it to be a set of innate building blocks such as features and categories and hierarchical maps, which all humans possess^2^. However, it is difficult to determine just which of these principles are innate: for example, cartographers such as Cinque and Rizzi (2009) have claimed that there are fine-grained restrictions on what kind of structures syntax can generate. But there is no widespread agreement on what the innate building blocks are, if any, with the potential sole exception of the syntactic operation Merge.

Indeed, more recent work in the Minimalist tradition of generative grammar is much less willing to commit to specifying precisely what the innate categories are. Chomsky et al. (2019) state that universal grammar is nothing more than a label for the difference in linguistic ability between humans and non-human animals. Furthermore, given the difficulty of explaining how such precise innate building blocks could have evolved, Minimalists have attempted to assume as little as possible. According to Bolhuis et al. (2014)'s “Strong Minimalist Thesis” (SMT) the language faculty is nothing more than general cognitive constraints plus the syntactic operation Merge to build recursive, hierarchical structure^3^.

In the second camp are, unsurprisingly, scientists who do not believe that there is a domain-specific module in the brain solely for language. Authors such as Christiansen and Chater (2015) claim that there is no such cognitive machinery: natural language can emerge merely from general cognitive constraints, at the very least for syntax. This is the claim that I would like to argue against in this article. Though there are different schools of thought in non-generative approaches to syntax, here I focus only on the alternative approach provided by Haspelmath (2020).

Haspelmath argues that the best way to study the unique human ability for language is *via* comparative methods: what he calls “g-linguistics” rather than “p-linguistics” which is the study of the grammar of a particular language. This is because much of the properties of any particular language could be historically accidental. He rightly notes that many authors—for instance (Chomsky, 1965; Lyons, 1977; Langacker, 1987; Grice, 1989; Jackendoff, 2002; Goldberg, 2005)—who made general claims on language did not do so based on comparative data. For our purposes, I need not take a side here: I will concur that the best way to study language is *via* comparative methods^4^. Even if he is right, I will argue that one can provide comparative evidence in favor of an innate machinery for syntactic structure.

As mentioned previously in section 1, under Haspelmath's comparative approach, we have two options: either there are innate building blocks that provide a rigid blueprint for languages, or the properties of each language are due to convergent cultural evolution^5^. He rightly notes that the approach which assumes innateness stumbles onto Darwin's problem: how could an innate blueprint have evolved within a million years, or potentially even less? It would preferable from an evolutionary standpoint to suppose that there are no innate building blocks, if possible. He instead proposes that the alternative is more likely: such crosslinguistic similarities arose due to convergent cultural evolution. Let us now see what he means; take, for instance, the following quote from Haspelmath (2020), in which he states his idea very clearly:

Just as nobody doubts that the cross-cultural existence of similar kinds of houses, tools, weapons, musical instruments and governance structures (e.g., chiefdoms) is not due to a genetic blueprint for culture but to convergent cultural evolution, there is also no real doubt that many similarities in the words of languages are due to cultural similarities and need no biological explanation. For example, many languages in the 21st century have short words for mobile phones, and these can be created in different ways (by abbreviating longer terms, e.g., Polish komórka from telefon komórkowy, by using a brand name, e.g., Natel in earlier Swiss German, or even letter abbreviations like HP in Indonesian, for hand phone).

At first glance, this approach appears to be much too rudimentary to derive more complicated syntactic generalizations across different languages. But section 5 will provide some methods to do so. Regardless, the rationale behind this approach is Darwin's problem: it is theoretically preferable to avoid positing innate building blocks if possible. By Occam's razor, if two theories make all the same predictions, we ought to prefer the one with the fewer assumptions. And assuming an innate blueprint would no doubt be far more costly than assuming mere functional explanations for syntactic generalizations. After all, according to Haspelmath, compelling crosslinguistic evidence has not yet been presented in favor of an innate building blocks. In the next two sections, I will attempt to do just so: there are crosslinguistic generalizations which are too fine-grained to be derived *via* reference to cultural evolution.

## 3. Introduction to Cartography

It is uncontroversial that the syntactic structures generated by human language use are complex. The goal of the cartographic enterprise in modern generative syntax is to draw highly detailed maps of these structures—as precise and as detailed as possible. As Cinque and Rizzi (2009) point out, under this conception of cartography, it is more of a research topic rather than a theory or hypothesis that attempts to determine what the right structural maps are for natural language. Although people may not agree on what the right map is, or even the right order of the projections on the map, Cinque & Rizzi still think that this shows the question is a legitimate one for modern syntactic theory.

Chomsky (1986)'s extension of X-bar theory to the CP-IP-VP structure of the clause was the critical step in allowing the advent of the cartographic program. This enabled syntacticians to conceive of clauses and phrases as made out of functional projections—these are heads like C (the head of the complementizer phrase, CP), I (the head of the inflectional phrase, I), and D (the head of the determiner phrase, D). But once these functional heads were added to the generative theory, it soon became clear that the same kind of evidence in favor of their existence also supported the existence of many more functional projections.

This is precisely what Pollock (1989) accomplished in his seminal paper on the I domain, arguing that I is not a unitary head but rather a domain made up of many functional heads—one for agreement, one for tense, and so on. Larson (1988) extended Kayne (1984)'s binary branching hypothesis to make similar arguments for the splitting of V into more functional projections. Finally and most importantly, as Rizzi (1997) has proposed, the functional projection C is not in fact just one functional projection, but it is a highly complex domain made out of many functional projections, each with a specific role.

I would now like to discuss the first piece of comparative evidence that has been provided in favor of cartography. Cinque (1999) sought to argue for the existence of a highly detailed and ordered universal hierarchy for clausal functional projections based on crosslinguistic data from several different languages, each of which are from different language families. This appears to be at odds with traditional analyses of adverbs in which they are adjoined with relative freedom and flexibility. But Cinque shows that they do not appear to have such freedom.

To be more specific, Cinque argues that clauses are made up of many functional projections which are ordered, and into each of those functional projections, an adverb can be inserted. If there is no adverb, then the functional projection is still present but simply not filled. This idea was first argued for by, I believe, Alexiadou (1997). But if there are multiple adverbs in a sentence, it is likely that they have to be ordered in some way—depending on the kind of adverb. Here is the order of adverbs that Cinque ends up with, based on his survey:^6^


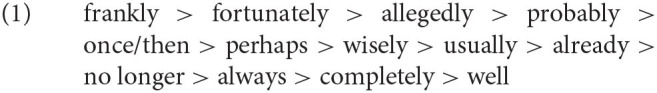


Let us now see some concrete examples, starting with English. Suppose we have a sentence with two adverbs: *any longer* and *always*, and they both appear before the verb. What we find is that the adverb *any longer* must precede the adverb *always*^7^:


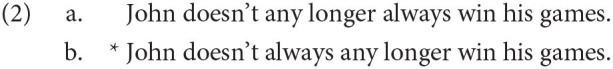


We find that this order is attested in Italian, as well, in addition to the several other languages that Cinque discusses:


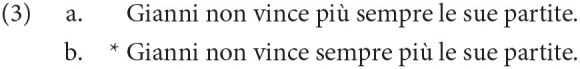


Another example is the ordering of what Cinque calls pragmatic adverbs like *frankly* over what Cinque calls illocutionary adverbs like *fortunately*. In Italian, what we find is that in a sentence with both adverbs, the pragmatic adverb must precede the illocutionary adverb, as in (4a)-(4b). Similar facts follow for the English translations as well: the English translation in (4a) is significantly preferable over the one in (4b), although the intuition may not be as strong as in Italian.


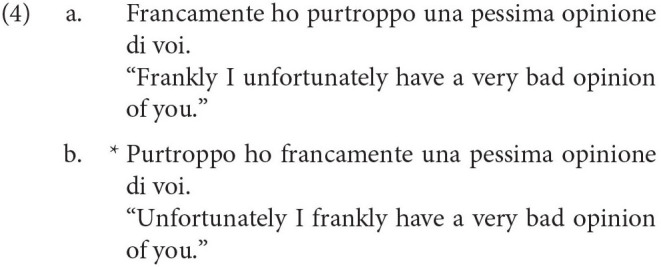


Cinque tests the ordering in (1) in many different languages: in addition to Italian and English, he also tests Norwegian, Bosnian/Serbo-Croatian, Hebrew, Chinese, Albanian, and Malagasy. He comes to the same conclusion in each of these languages. That such fine ordering is attested in all of these languages belonging to different language families appears to be strikingly coincidental, if not for the potential presence of innate building blocks—or some cognitive constraints from which these patterns could be derived.

We have just seen Cinque (1999)'s evidence that there is very fine ordering between numerous adverbs, and this ordering is attested from language to language in different language families. It is exceedingly unlikely that the ordering of adverbs seen in (1) above can be derived *via* reference to functional methods, or cultural evolution. The only alternative in that case, as Haspelmath suggests, is that there are innate building blocks that guides the order in which adverbs are present in syntactic structure.

There appears to be a problem that puts cartography at odds with the Minimalist framework developed by Chomsky (1995). This is one that Haspelmath (2020) briefly mentions as well (p. 7). There seems to be a tension between the very simple mechanism that drives the formation of recursive structure for Minimalists—that is, Merge—and the very fine and complex cartographic representations that are argued to be innate in the language faculty. Cinque and Rizzi (2009) suggest that there is no inherent conflict between the two viewpoints: they believe that the tension is merely “the sign of a fruitful division of labor.” They describe how the two approaches might come together very clearly in the quote below:

Minimalism focuses on the elementary mechanisms which are involved in syntactic computations, and claims that they can be reduced to extremely simple combinatorial operations, ultimately external and internal Merge, completed by some kind of search operation (Chomsky's Agree) to identify the candidates of Merge. An impoverished computational mechanism does not imply the generation of an impoverished structure: a very simple recursive operation can give rise to a very rich and complex structure, as a function of the inventory of elements it operates on, and, first and foremost, of its very recursive nature.

Thus, I believe that cartography is not in conflict with a weaker version of Minimalism, which is more of a philosophy than a thesis: the fewest number of innate building blocks that are necessary ought to be assumed in our theory. But the most natural way to understand cartography is in terms of an innate blueprint. It appears that any account which assumes an innate blueprint for syntactic structure in the language faculty is at odds with Bolhuis et al. (2014)'s SMT, which we discussed previously.

But what is the nature of this blueprint?^8^ The functional hierarchies could be encoded in a certain order, such as (1) directly onto the language faculty. This possibility can be immediately dismissed via Darwin's problem noted by Haspelmath.^9^ Furthermore, as Chomsky et al. (2019) note, there is no conceivable evidence that a child would be able to infer fine hierarchical details from experience. It would be preferable to suppose that the hierarchy in (1) may not be directly encoded but could be derived from more general and basic principles and properties, which are a part of the computational machinery of the human language faculty, which Ernst (2002) attempts to do by reference to their semantics. Several intermediate possibilities may exist as well. The blueprint must thus be more minimal than a complex order of functional projections. The job of the cartographer, then, is to find the correct maps and then trace them to more general properties.

## 4. Further Cartographic Generalizations

In this section, my goal is threefold. I will first introduce the reader to the basics of the complementizer domain in generative linguistics, and then present (Rizzi, 1997)'s cartographic approach. I do so in order for the reader to be able to more clearly understand recent crosslinguistic evidence in favor of Rizzi (1997)'s cartographic approach, from Sabel (2006) and Satık (2022). But ultimately, as I will discuss further at the end of the section, it is immaterial that I am taking for granted a generative framework here. The crosslinguistic generalizations I will discuss here still exist and need to be accounted for—whether or not one assumes a generative framework. The only reason I am introducing the basics of generative grammar is so that the reader can understand the background that drove the finding of these comparative patterns.

### 4.1. The Complementizer Domain in Generative Grammar

Let us start now with an introduction to the properties of the complementizer (C) domain in the CP-IP-VP conception of clauses in generative grammar. A *complementizer* is a word or morpheme that marks an embedded clause functioning as a complement—for example, a subject (***That*** *the world is flat is false*) or an object (*Scientists believe* ***that*** *there may be life on Venus*). In both cases, the complementizer is *that*, which is the complementizer that is associated with finite clauses in English. However, there is reason to believe that the C domain has other properties, such as containing wh-words^10^. This is referred to the “doubly-filled COMP filter” in the generative literature, which excludes the complementizer *that* co-occurring with a wh-element. The fact that they are in complementary distribution indicates that they are related to each other, as seen in example (5)^11^:


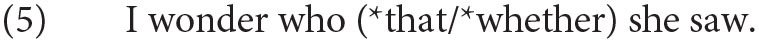


A complementizer that is often associated with infinitival clauses in English is *for*. Although *to* is sometimes treated as an infinitival complementizer [ex. (Pullum, 1977)], here I follow Pullum (1982) and Pollard and Sag (1994) in assuming that it is not a complementizer, but rather more like a verb. Their arguments are based on VP-ellipsis; instead, I would like to simply note that *to* can co-occur with wh-words in English, as in the sentence *I know what to eat* which is completely natural. The fact that this sentence differs strongly in acceptability in comparison to (5) indicates that *to* may not be a real complementizer.

There are other properties that are often associated with the C-domain in addition to the presence of complementizers and wh-words. It is well known that English allows the fronting of topics, for example in *I ate the cookie* the object can be fronted in certain contexts, leading to the sentence *The cookie, I ate*. In generative grammar, the location of sentence topics is driven by the process of *topicalization*—which is a mechanism in generative syntax that moves an expression to the front of a sentence to establish it as a sentence topic. I do not need to assume that this takes place *via* movement. Regardless, the location of topics is also thought to be in the C domain. Note that a sentence with both a topic and a *wh*-element is significantly degraded, at least in English, as demonstrated below in (6c), which has both:


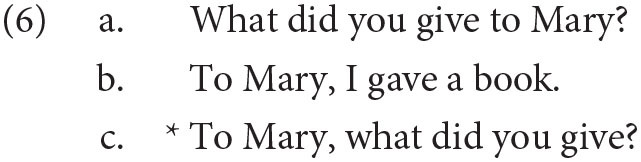


The presence of fronted expressions due to focus, which is due to a process called *focalization* in generative grammar, which for similar reasons is also thought to take place to a position in the C domain:


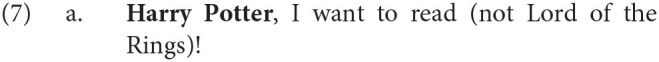


To recap, under a generative framework, we have seen that the complementizer domain is not responsible for just the presence of *that*, but several other properties as well. In the previous section, I briefly mentioned a few works which argued in favor of splitting the IP and VP domains into further syntactic projections. The goal of Rizzi (1997) is to argue that the C domain is also similarly set up: if we only had a single functional projection, C, it would be impossible for it alone to be responsible for all of the aforementioned properties involving wh-words, complementizers, topics, and focalized elements.

In addition to this, Rizzi gives empirical evidence that the C domain itself is split up, by showing that there are two different kinds of complementizers. In Italian, for example, we see in (8) below that it is impossible to place topics in a position to the left of the high complementizer *che* (which Rizzi calls a finite complementizer), but it is possible to place topics to its right^12^.


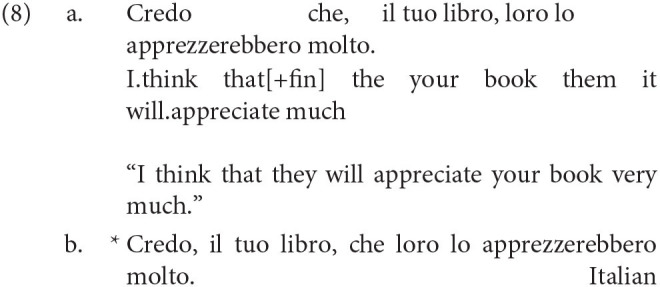


This contrasts with the behavior of the low complementizer *di* (which Rizzi calls a nonfinite complementizer), which only allows one to place topics to its left in (9).


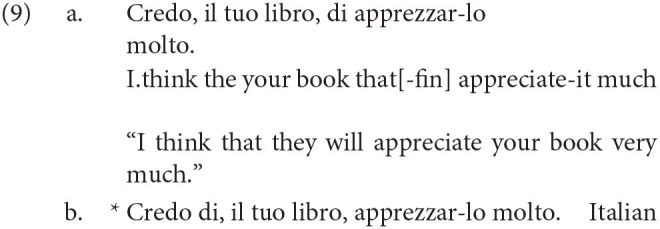


Regardless of whether or not one buys the generative enterprise, there does appear to be two kinds of complementizers—one which necessarily precedes topics, and one which necessarily follows them. This will be crucial for the upcoming empirical generalization that we will discuss in the next subsection.

Under a generative framework, what we need is a system of *ordered* projections in the C domain which will get us the right order. It is impossible for a single functional projection in the C domain, to account for all of these properties: topics, foci, wh-elements, high and low complementizers, and so on. There is more than just a single projection for complementizers; we need two which contain the projections for topic and focus sandwiched between them^13^. This is what will appear to be the case in the empirical survey of infinitives that will be presented in the next subsection.

There is reason to believe that there are many more projections than what Rizzi (1997) has initially claimed, and the number of functional projections has indeed increased in works since then such as Haegeman (2012). For our purposes, I will briefly discuss only the additional projections which are relevant—WhyP and WhP in particular. Starting with WhyP, Shlonsky and Soare (2011) notes that there is a contrast between finite and nonfinite clauses in English; the former allows *why* but the latter does not:


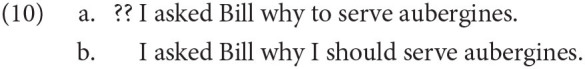


This is despite the fact that English allows wh-infinitives, such as *I know what to eat*, indicating that “why” and other wh-elements need to be ordered differently in Rizzi's hierarchy. I will conclude with the following hierarchy of ordered functional projections, following Shlonsky and Soare (2011)^14^:


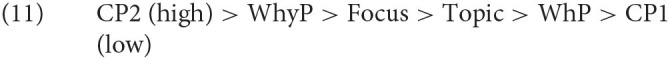


This sets the stage to make purely theory-neutral and empirical generalizations in the next subsection. The generalizations, as we will see, are true regardless whether one believes in the generative approach. But I believe that the hierarchy seen in (11) must be present in one form or another to make sense of the upcoming crosslinguistic generalizations.

### 4.2. The Left Periphery of Infinitives

The comparative evidence present in the literature regarding Rizzi's cartography concerns infinitives, so an introduction into the left periphery of infinitives is necessary prior to presenting what appear to be crosslinguistic generalizations. I would like to start by noting that infinitives differ in terms of the properties of the C domain they allow. Some allow topics, some allow wh-words, some allow why, some allow focalized elements. However, crucially, according to Sabel (2006) and Satık (2022), the properties that a language allows is *predictable* from Rizzi's cartography. This is what will set the stage for the argument that an innate building blocks is present in the language faculty. Without such innate properties—perhaps a blueprint like a map—such properties would simply not be predictable.

Adger (2007) notes a contrast between English and Italian infinitives; topics are not allowed at all in English infinitives, whether or not the nonfinite complementizer *for* is present. We previously saw that Italian infinitives allowed topics in example (9) above. In other words, Italian infinitives allow topics, while English infinitives never do.


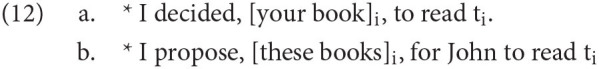


Yet, both English and Italian allow wh-words in their infinitives:


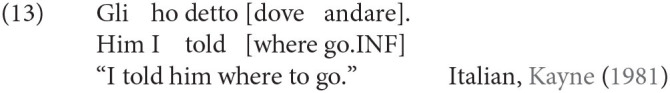


Other languages like Hindi do not; the sentence below is ungrammatical:


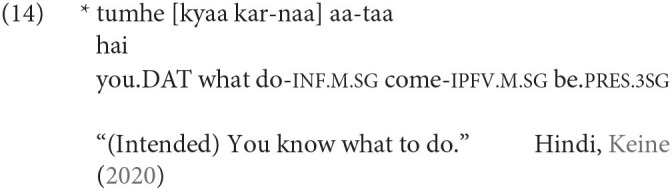


Under a generative framework, this data indicates two things. First, the left periphery of the infinitive is truncated. Second, languages differ as to the degree of truncation.

What Sabel (2006) finds, based on a study of several Germanic, Romance and Slavic languages is that whether a language has a nonfinite complementizer is predictable based on whether it has wh-infinitives. That is, if a language allows wh-elements within its infinitive, then it must also have infinitival complementizers. This, Sabel claims, is simply because the presence of wh-elements necessarily implies the existence of the C domain in the infinitive of that language. This is the first instance of comparative evidence in favor of Rizzi's approach. This is a completely theory-neutral and empirical observation: one need not assume a generative framework to come to this conclusion.

I extend Sabel's observation to several other properties of the infinitival left periphery and conduct a more rigorous crosslinguistic sample in Satık (2022), based on the aforementioned properties of the C domain such as the presence of topics. Based on a survey (presented in Table 1 below) of 26 languages belonging to many different language families, I conclude that the following crosslinguistic generalizations are true.


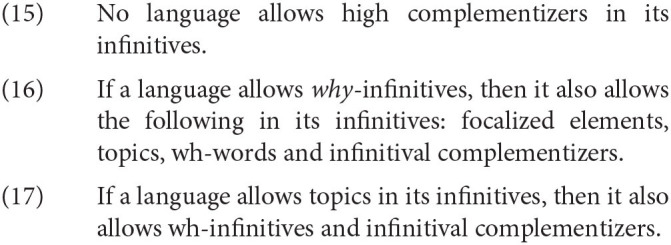


**Table 1:** The column “High C” is to state whether the language allows a high complementizer in its infinitives or not.

**Language**	**High C**	**why**	**focus**	**topic**	**wh-words**	**Low C**	**Source**
Bangla	✗	✗	✗	✗	✗	✗	Dasgupta (1982)
Catalan	✗	✗	✗	✓	✓	✓	Villalba (2009)
Danish	✗	✗	✗	✗	✗	✓	Satık (2022)
Dutch	✗	✗	✗	✗	✓	✓	van der Auwera and Noel (2011)
English	✗	✗	✗	✗	✓	✓	Satık (2022)
E. Portuguese	✗	✗	✗	✗	✓	✓	Barbosa (2001)
French	✗	✗	✗	✗	✓	✓	Barbosa (2001)
German	✗	✗	✗	✗	✗	✗	Sabel (2006)
Hebrew	✗	✓	✓	✓	✓	✓	Shlonsky (2014)
Hindi	✗	✗	✗	✗	✗	✗	Keine (2020)
Hungarian	✗	✓	✓	✓	✓	✓	Szécsényi (2009)
Ibibio	✗	✗	✗	✗	✗	✗	Doherty (2016)
Icelandic	✗	✗	✗	✗	✗	✓	Thráınsson (1993)
*Irish*	✗	✗	✗	✓	✗^f^	✓	Satık (2022)
Italian	✗	✗	✗	✓	✓	✓	Satık (2022)
*Jordanian Arabic*	✗	✗	✗	✗	✗	✗	Al-Aqarbeh (2011)
*Mandarin*	✗	✗	✗	✗	✓	✓	Ussery et al. (2016)
Middle English	✗						Satık (2022)
Norwegian	✗	✗	✗	✗	✗	✓	Faarlund (2015)
Old Norse	✗						Faarlund (2015)
Old Swedish	✗						Kalm (2016)
Russian	✗	✓	✓	✓	✓	✓	Satık (2022)
Serbian	✗	✗	✗	✗	✗	✗	Satık (2022)
Spanish	✗	✗	✗	✓	✓	✓	Villalba (2009)
Swedish	✗	✗	✗	✗	✗	✓	Kalm (2016)
Turkish	✗	✗	✗	✗	✗	✗	Kornfilt (1996)

*The column “Why” is to state whether the language allows "why" in its infinitives or not. The column “Focus” is to state whether the language allows focalized elements in its infinitive or not. The column “Topic” is to state whether the language allows topics in its infinitive or not. The column “wh-words” is to state whether the language allows wh-words in its infinitive or not. The column “Low C” is to state whether the language allows low complementizers in its infinitive or not. The column “Source” is to state where the information was obtained for a given language. Languages which do not have a clear finite-nonfinite contrast are marked with italics in the table. Given that Middle English, Old Norse and Old Swedish cannot be investigated further, I have mostly left the entries for these languages blank. What is clear in the literature is that these languages do not allow high complementizers in infinitives*.

Here are some examples of how the generalizations in (15)–(17) work. For example, given that we mentioned previously that Italian infinitives allow topics, (17) predicts that Italian should allow have wh-words in infinitives. This prediction is borne out. The other languages that allow topics in infinitives, such as Hungarian, Hebrew, Russian, and Catalan, also allow wh-infinitives^15^.

Another example is the fact that no language allows high complementizers in its infinitives. For instance, let us consider Icelandic, a language which allows a complementizer with the phonetic form *ad* in both its finite and nonfinite clauses. Thráınsson (1993) notes a crucial difference between *ad* in infinitives and in finite clauses, however. What we find is that while *ad* behaves as a high complementizer in finite clauses as in (18a), given that a topic may follow *ad*, this is impossible in a nonfinite clause; a topic cannot precede nor follow *ad*, indicating that *ad* cannot behave as a high complementizer in a nonfinite clause. This is a property that appears to remain stable crosslinguistically among languages with infinitives.


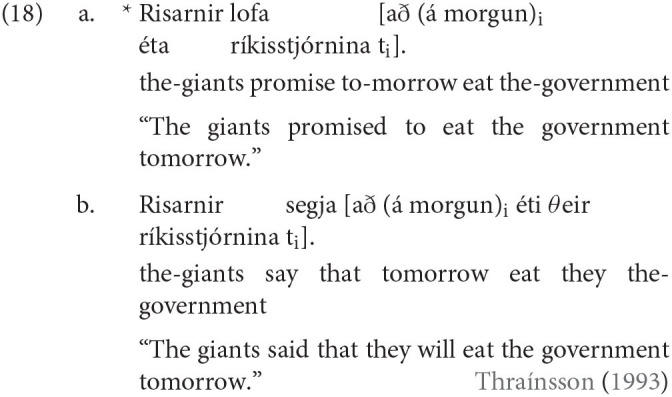


Ultimately, the observations in (15)–(17) are theory-neutral observations. But I argue in the next section that such generalizations cannot plausibly be captured by assuming that linguistic patterns are driven by convergent cultural evolution, or functional methods of explanation. I believe this indicates the truth of Rizzi's cartographic structure of the C domain.

## 5. Functional Explanations?

As discussed previously in section 2, Haspelmath (2020) proposes that similarities across languages arise in order to serve the needs of their speakers. Such patterns, he claims, are much more likely to have risen *via* convergent cultural evolution rather than innate building blocks that guide linguistic outputs. Instead of generative explanations for syntactic phenomena, there are “functional-adaptive” explanations to crosslinguistic patterns in word-class and word order^16^. Let us see an example of how such an explanation might proceed.

The idea behind functional explanations to linguistic phenomena, which goes back to Saussure (1916), is that speakers themselves are responsible for language structure for their use. Language is a tool which speakers change to better serve their tasks such as conveying meaning or contextual information. Saussure saw linguistics as a social science, according to which languages are a social construct. Take, for instance, the following observation made by Dryer (1988)^17^. According to Dryer's sample of 325 languages, 227 of these, or 70%, place the negative before the verb. For example, in English we would say *I did not take out the trash*, where the negative precedes the verb *take*. Dryer's functional-adaptive explanation is as follows. Negative morphemes are a crucial part of the message of a sentence and, hence, carry a large communicative load. For if a speaker does not hear the negative morpheme in a sentence, they will understand just the opposite of the intended meaning of the sentence. Dryer suggests that one way to decrease the chance of this occurring is to place it before the verb, as delaying would increase the chance of a misunderstanding.

With the methodology established, we can now determine whether it is able to come up with an explanation of our cartographic generalizations. Let us start with the hierarchy of adverbs created by Cinque (1999), which noted that the same hierarchy is found in several different languages, each of which belong to a different language family:


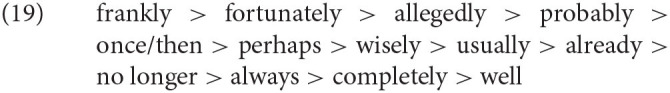


Although functional explanations of certain phenomena such as the crosslinguistic ordering of negative morphemes are both plausible and possible, this is much less likely to be the case with the ordering in (19). The ordering is too fine-grained to have risen from convergent cultural evolution, given that there is no apparent functional advantage to ordering adverbs in this way. For instance, consider the difference in ordering between *frankly* and *fortunately* in Italian, from Cinque (1999) below:


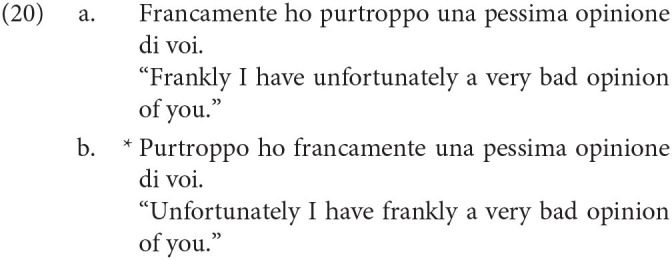


It is exceedingly unlikely that speakers of Italian would use these two adverbs frequently enough to make any decisions on how they ought to be ordered. Furthermore, even if speakers of Italian did use such adverbs frequently, what would be a possible functional explanation for the unacceptability of (20b)? For instance, there is no problem of miscommunication if one were to utter (20b). Even if adverb ordering facts arise from semantics as Ernst (2002) claims, issues still arise. What communicative advantage is there to order propositional adverbs before event ones? And how would a child be capable of learning the complicated semantic categories which adverbs fit into? If Haspelmath is right that there are only two sources of explanation for crosslinguistic patterns, then the only alternative that remains to us is that the ordering in (19) is innate.

I will now repeat some of the cartographic generalizations regarding infinitives we have seen in the previous section:


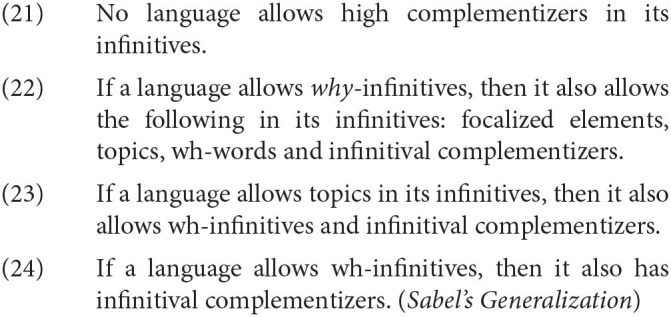


Once again, there is a great deal here that has to be explained *via* reference to functional-adaptive methods under the cultural approach. For example, there is no functional advantage to banning a certain kind of complementizer from infinitives—indeed, it seems to be innocuous^18^. But due to reasons of space, I can only discuss one problem in detail. Take, for instance, the difference in acceptability between normal wh-infinitives and why-infinitives in English:


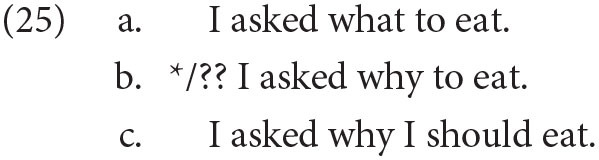


What would be the functional explanation for the unacceptability of (25b), while (25a) is acceptable? (25b) in languages such as Hebrew, Russian and Hungarian is acceptable, as seen in Table 1. As such, it appears that (25b) is perfectly semantically well-formed; the intended interpretation is given in (25c), which is acceptable given that it is finite. One would need a functional explanation of ruling out *why* in the infinitive of many different languages, which does not seem to be available: there is no apparent increase in communicative load in (25b) compared to (25c). In each of these cases, the burden of proof is on the functionalist to explain how such fine distinctions in acceptability can arise *via* reference to cultural evolution.

Furthermore, the cartographic and functional approaches make different predictions. According to a cartographic approach, the difference arises because the left periphery of the infinitive in English is too deeply truncated to allow the presence of *why*. And according to Shlonsky and Soare (2011)'s cartographic ordering of *why* over topics, focalized elements, wh-words, we would expect that languages with *why*-infinitives allow focalized elements and topics within infinitives. This prediction is borne out. A functional approach is not able to predict these generalizations, given that there could be no innate blueprint.

I have argued that purely functional explanations are not sufficient. Two anonymous reviewers make a very similar suggestion regarding the cartographic generalizations discussed here, agreeing that it is not the case that *only* cultural evolution is at play here. But rather than claiming that only innate linguistic properties are responsible for these generalizations, one might consider a feedback loop between adaptive cultural and biological changes that would potentially lead to the creation of some innate linguistic properties, perhaps a blueprint, that would be able to account for the generalizations discussed in this article.

It is important to point out that first and foremost, this argument “bites the bullet,” so to speak: it admits that there are some innate language-specific properties in the brain, which is the goal of this article^19^. This is desirable. Regardless, an account along these lines is given by Progovac (2009), on deriving movement constraints in the generative grammar framework. Ross (1967) notes, in his seminal dissertation, that there are many types of “exceptional” syntactic environments which do not allow movement out of them, calling these *islands*. Note, for instance, the sharp difference in acceptability between the sentences below. Coordination structures such as *A and B* are an example of an island:


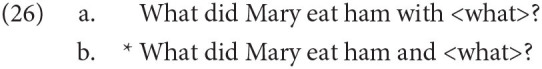


From an evolutionary perspective, the existence of islands is puzzling. As Lightfoot (1991) claims, it is difficult to see how constraints on movement could have led to “fruitful sex.” How could a grammar with islands be selected over a grammar without? For reasons such as these, Berwick and Chomsky (2016) assume that syntax did not evolve gradually, but rather, it was the product of a single mutation.

This is a pill that is hard to swallow, and indeed even more difficult to do so given the fine cartographic generalizations discussed here. Progovac proposes a way of deriving islandhood *via* gradual evolution, similar to the feedback loop discussed above. She takes islandhood to be the default state of syntax, where movement itself is seen as an exceptional operation. She notes that movement itself is only available out of a subset of complements, which form a natural class—but the set of islands do not form one, as islands range from adjuncts and conjuncts among other things. For Progovac, movement evolved from a proto-syntax with small clauses and one-word utterances. The evolution of subordination and movement by the need to embed multiple viewpoints within each other, for which coordination and adjunction did not suffice. For example, in Progovac's examples below, only in (27c) can a person's knowledge about another be reported:


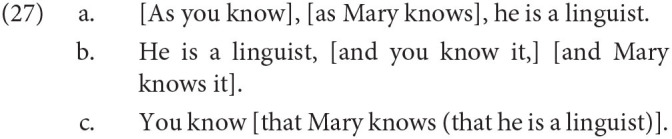


I am not opposed to this kind of feedback loop or gradual evolution of syntactic properties. However, there are a few crucial differences between the generalizations discussed in this article and generalizations like islandhood. First, there is a sense in which islandhood is a (partly) theory-internal phenomenon, defined in terms of theoretical terms such as movement. This has the risk of being a non-starter for scientists who do not accept the generative framework. By contrast, the generalizations proposed here are not theory-internal, and they can be described in completely descriptive terms, with no reference to generative operations such as movement.

More importantly, cartographic generalizations are not prima facie amenable to a solution like Progovac's for islandhood. While the development of subordination and movement might indeed lead to “fruitful sex,” it is unclear how, for instance, adverb ordering could. Speakers rarely, if ever, use the adverbs *any longer* and *always* together in a sentence—it is difficult to imagine that such fine ordering could lead to natural selection. The hope is that in the distant future, general, potentially semantic, principles that lead to this fine ordering will be found, that would be amenable to an explanation in terms of a feedback loop.

## 6. Concluding Remarks

We have just seen multiple examples of cartographic generalizations that appear to be attested crosslinguistically. Such fine ordering, seen in language after language belonging to many different language families, is very unlikely to be a cultural property, as Cinque and Rizzi (2009) note—there is no conceivable functional advantage for them. If Haspelmath is right, this entails the stipulation of innate machinery for syntactic structure in the human brain.

One particularly difficult question remains that cannot yet be answered, however. Haspelmath (2020) rightly notes that it appears unlikely for an innate blueprint to have evolved within a million years or less. By contrast, prima facie, it does seem more plausible that a grammatical feature evolved culturally over just a few generations. And yet, the truth of the data indicates that there is a fine ordering of syntactic categories. And it is unlikely for such fine ordering to have evolved culturally. Though the evidence seems to indicate the presence of the blueprint, its nature and how it evolved remain elusive.

As Cinque and Rizzi (2009) point out, there must be principles that determine the hierarchical structure seen language after language and allow children to obtain the right order during language acquisition. Cinque and Rizzi suggest that certain elements of the hierarchy can be derived their semantic properties: in the case of focus and topic, for example. Ernst (2002) attempts to derive the ordering of adverbs via their semantics. Rizzi (2013) builds on this reasoning further, by providing a possible explanation of the crosslinguistic asymmetry between the ordering of topic—which can be reiterated in many languages—while left-peripheral focus cannot.

However, other parts of the hierarchy cannot be semantic and must be purely syntactic; that high and low complementizers exist is clear, and they have no semantic function other than identifying the clause as a complement. But *why* high and low complementizers exist and the source of the fine ordering between the different elements of the complementizer domain will remain a mystery for the foreseeable future.

My goal in this paper has been relatively modest: while I am not able to answer where exactly cartographic generalizations come from, I tried to show that there are at least some generalizations which cannot have a purely functional explanation. Much remains open to research. However, what I hope to have shown is that there are crosslinguistic generalizations made in a generative framework that ought to be taken seriously, and cognitive scientists may end up needing to assume the presence of an innate hierarchy of syntactic categories.

## Author Contributions

DS conceived and wrote the manuscript.

## Conflict of Interest

The author declares that the research was conducted in the absence of any commercial or financial relationships that could be construed as a potential conflict of interest.

## Publisher's Note

All claims expressed in this article are solely those of the authors and do not necessarily represent those of their affiliated organizations, or those of the publisher, the editors and the reviewers. Any product that may be evaluated in this article, or claim that may be made by its manufacturer, is not guaranteed or endorsed by the publisher.

## Footnotes

^1^An anonymous reviewer points out that this example may not be a perfect analogy, because the position and structure of hearts and kidneys is relatively rigid, whereas languages are subject to far more optionality. This is true, but I would like to point out, here, that the *parameters* of variation for human language are rigid. For example, languages need not have high complementizers or infinitives (see section 4), but if they have both, then high complementizers cannot co-occur with infinitives.

^2^Although Barsky (2016) states that universal grammar would suggest that all languages possess the same set of categories and relations, this is not quite right. Chomsky, among many others, note that what universal grammar suggests is merely the innate capacity for humans to *acquire* natural language. This is the same point that has been made by Chomskyan linguists in response to Everett (2005)'s arguments against the existence of universal grammar based on the apparent lack of recursion in Pirahã. Even if Pirahã does not have recursion, this does not mean that the Pirahã people do not have a language faculty: this is evidenced by the fact that they are able to learn Portuguese. This is all that Chomsky's hypothesis predicts, and it does so correctly.

^3^If it turns out that the cartographic generalizations presented in this article cannot be derived without the need for innate maps for syntactic structure, this view would be at odds with the cartographic account that I assume and defend in this article. Although Cinque and Rizzi (2009) suggest that there is inherently no tension between cartography and Minimalism, in my view this is at odds with the SMT, which is a stronger version of Minimalism.

^4^See, for instance, Mendívil-Giró (2021) who argues against this claim. Given that this is tangenital to my goal in this article, I will not pursue this here.

^5^Haspelmath (2020) briefly notes that a third option is a possibility for similarities in crosslinguistic patterns: historical accidents. An anonymous reviewer suggests seeing this option as more of a product of certain historical laws, or laws of evolution, rather than accidents. Although I do not wish to deny the existence of this possibility entirely, I would like to note that it does not appear to fare any better at handling the cartographic generalizations presented in this article. For crosslinguistic similarity to have evolved in a species, it needs to have exerted a strong enough evolutionary pressure. It is difficult to imagine that the very fine ordering of adverbs according to Cinque (1999), for instance, could exert enough evolutionary pressure to make such fine ordering arise in the human species *via* evolution.

^6^I will be unable to present extensive evidence for the hierarchy in this paper due to reasons of space. The reader is referred to Cinque (1999) for further evidence.

^7^There is one little catch with this data. Notice that the sentence *John doesn't always win his games any longer* is acceptable, in which *always* appears to precede *any longer*. This is also possible in Italian, according to Cinque, but only if *any longer* is emphasized. Without emphasis, it is not possible. As Cinque notes, appearances are deceiving: one could suppose that it involves movement of the adverb from its initial position.

^8^I am grateful to an anonymous reviewer for helpful comments with this discussion.

^9^See Bobaljik (1999) for an argument that such an account also leads to a paradox.

^10^Of course under the generative framework, this process involves the syntactic operation of movement, and hence is called *wh*-movement. But here I am abstracting away from generative assumptions as much as possible.

^11^This is with the exception of Belfast English, see Henry (1995).

^12^I note in Satık (2022) that there is considerable crosslinguistic evidence that the distinction between high vs. low complenentizers is not unique to Italian. It is widely attested crosslinguistically, in other Romance languages [such as Spanish, according to Villa-Garcia (2012), in the Scandinavian languages (Larsson, 2017), in the Niger-Congo language Lubukusu (Carstens and Diercks, 2009) and even in English (Haegeman, 2012)].

^13^For reasons of space, I will not present Rizzi's arguments for ordering topics with respect to focalized elements; although this is not strictly speaking correct, I will assume that focalized elements are always ordered prior to topics for simplicity. Rizzi (1997) orders the projection for topics recursively, such that they can occur before or after focus, or other projections in the C domain as well. But they must necessarily be sandwiched between high and low complementizers. Furthermore, Rizzi dubs the heads responsible for high and low complementizers as ForceP and FinitenessP respectively. Again, I abstract away from these names and name them CP2 and CP1 respectively for simplicity.

^14^This is not quite right; Shlonsky and Soare (2011) assume that WhyP is in fact called InterrogativeP and it can also house words like *if* . They argue that it is higher than FocusP under Rizzi's theory. However, for simplicity, I have abstracted away from this term.

^15^The only apparent counterexample to one generalization is Irish, and it is marked on the table with the superscript letter “f.” It allows topics in its infinitives but does not allow wh-elements. This is not a true counterexample to my generalization, however. Irish wh-questions are well-known for being copula sentences; thus, this is in fact unsurprising, and the possibility of wh-questions in Irish nonfinite clauses is excluded for independent reasons.

^16^See, for example, Croft (2000); Haspelmath (2008), and Hawkins (2014).

^17^This observation was first made, as far as I am aware, by Jespersen (1917).

^18^Though such an observation has striking consequences on the nature of finiteness in the framework of generative grammar, for it indicates that finiteness may be defined as a matter of clause size. See, for example, Müller (2020), Pesetsky (2021) and Satık (2022). As an anonymous reviewer points out, this contradicts certain approaches in which all projections are present even if not overtly realized—even in nonfinite clauses. See Wurmbrand and Lohninger (2019) for arguments against such approaches.

^19^This would directly contradict (Berwick and Chomsky, 2016)'s claim that the syntactic operation Merge arose from a single genetic mutation, at least when assumed in conjunction with Bolhuis et al. (2014)'s SMT, which states that the language faculty is nothing but Merge.
